# Cell migration inducing hyaluronidase 1 promotes growth and metastasis of papillary thyroid carcinoma

**DOI:** 10.1080/21655979.2022.2074110

**Published:** 2022-05-11

**Authors:** Min Zhou, Wei Hua, Yulan Sun

**Affiliations:** Department of Pathology, The First Affiliated Hospital of Harbin Medical University, Harbin, China

**Keywords:** CEMIP, metastasis, papillary thyroid carcinoma, PDK4, STAT3/AKT/NF-κB

## Abstract

Cell migration inducing hyaluronidase 1 (CEMIP) mediates catabolism of hyaluronan, and participates in the cell metastasis, invasion, and motility. Dysregulated CEMIP expression was associated with progression and prognosis of tumors. The role of CEMIP in papillary thyroid carcinoma (PTC) remains unknown. Our study showed that CEMIP was upregulated in both tissues and cells of PTC. Silencing of CEMIP reduced cell proliferation and suppressed migration and invasion of PTC. Protein expression of phosphorylated STAT3 (Signal Transducer And Activator Of Transcription 3) (p-STAT3), AKT (p-AKT) and p65 (p-p65) were decreased by CEMIP silencing in PTC cells. Pyruvate dehydrogenase kinase 4 (PDK4) over-expression attenuated CEMIP silencing-induced decrease in p-STAT3, p-AKT and p-p65. Silencing of CEMIP-induced decrease in cell proliferation and metastasis in PTC were restored by over-expression of STAT3. CEMIP functioned as an oncogenic gene in PTC through PDK4-mediated activation of STAT3/AKT/NF-κB pathway.

## Graphical abstract

**Figure:**
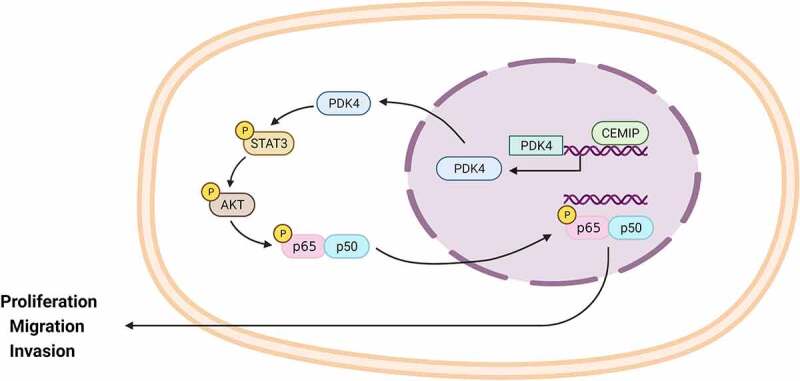

## Highlights

- Knockdown of CEMIP reduced cell proliferation and metastasis of PTC.
- CEMIP promoted PDK4-mediated activation of STAT3/AKT/NF-κB pathway in PTC.
- CEMIP was a potential target for PTC.

## Introduction

Thyroid cancer is the most common endocrine cancer characterized with slow tumor growth and low malignancy [1]. Highly rates of persistence and recurrence contribute to morbidity and incurability of thyroid cancer [1]. Papillary thyroid carcinoma (PTC) accounts for 85% of thyroid cancer [2]. Thyroid-stimulating hormone suppression, radioiodine and thyroidectomy therapies are the common clinical managements for PTC [2]. However, these managements are not effective for the metastatic tumors with high recurrence and poor prognosis [3]. Novel therapeutic targets are required to improve treatment of metastatic PTC.

Cell migration inducing hyaluronidase 1 (CEMIP) catalyzes catabolism of hyaluronan, and participates in regulation of hyaluronic acid-rich organ diseases, including ovary, testis, lung, skin, and brain cancers [4]. High CEMIP expression was related to poor prognosis of colon cancer [5] and reduction of CEMIP repressed colorectal cancer invasion and metastasis [6]. Over-expression of CEMIP promoted cell invasion and metastasis of ovarian cancer [7], and CEMIP protein secreted by tumor exosomes promoted brain cancer cell colonization and metastasis [8]. However, the role of CEMIP in PTC remains unknown.

Pyruvate dehydrogenase kinase 4 (PDK4), a key enzyme in mitochondrial respiration and glucose metabolism, participates in the ferroptosis sensitivity [9] and aerobic glycolysis [10] during the development of tumors. PDK4 was also involved in migratory, proliferation, and invasion of thyroid cancer cells [11]. CEMIP has been shown to enhance PDK4-mediated metabolic reprogramming to promote metastasis of prostate cancer [12]. However, the potential role of CEMIP/PDK4 in PTC progression remains unclear.

This study hypothesized that CEMIP was an oncogene in PTC. The effects of CEMIP on PTC cell progression were investigated, and the underlying mechanism of CEMIP-mediated PTC progression might provide target for PTC.

## Materials and methods

### Human specimens

Patients with PTC (N = 60) were recruited at The First Affiliated Hospital of Harbin Medical University. The tumor and normal tissues were collected via surgical procedures according to previous research [13]. This study was approved by the Ethics Committee of First Affiliated Hospital of Harbin Medical University (Approval No. IRB-AF/SC-04/01.0) and in accordance with those of the 1964 Helsinki Declaration and its later amendments for ethical research involving human subjects [14].

### Immunohistochemical analysis

Sections of PTC tissues were treated with 3% H_2_O_2_ and immersed in Tris-EDTA buffer before incubation with 5% dry milk. Antibody against CEMIP (1:500) was used to probe sections, and horseradish peroxidase-labeled secondary antibody was then used to incubate the sections. Sections were then treated with diaminobenzidine (Sigma-Aldrich, St. Louis, MO, USA), and measured under light microscope (Olympus, Tokyo, Japan) according to previous research [13].

### Cell culture and transfection

Human PTC cells, (TPC-1, KTC-1, CAL62, K1) and HTori-3, were cultured in RPMI-1640 medium with 10% fetal bovine serum (Gibco, Carlsbad, CA, USA) according to previous research [13]. shRNAs targeting CEMIP (sh-CEMIP#1 and #2) and the negative control (sh-NC), pcDNA-CEMIP, pcDNA-PDK4, pcDNA-STAT3 and negative control (pcDNA) were synthesized by GenePharma (Suzhou, China). TPC-1 and Cal62 were transfected with the shRNAs and pcDNAs by Lipofectamine 2000 (Gibco).

### Quantitative reverse transcription PCR

RNAs isolated from tissues and cells were reverse-transcribed into cDNAs. SYBR Green Master (Roche, Mannheim, Germany) was used for the RT-qPCR analysis of CEMIP with following primers: CEMIP (forward: 5’-GCTCTGGGATTTAAGGCAGC-3’ and reverse: 5’-ATTGGAGCCATGGACTGTGA-3’) and GAPDH (endogenous control; forward: 5’-ACCACAGTCCATGCCATCAC-3’ and reverse: 5’-TCCACCACCCTGTTGCTGTA-3’) according to previous research [13].

### Cell viability and proliferation assays

Two days post transfection, TPC-1 and Cal62 were seeded in 96-well plate, and incubated for 24, 48, or 72 hours. CCK8 (Cell Counting Kit-8) (Dojindo, Tokyo, Japan) was added and incubated for 2 hours, and absorbance at 450 nm was measured with Microplate Autoreader (Thermo Fisher, Waltham, MA, USA). To detect cell proliferation, TPC-1 was seeded in 6-well plate, and cultured for another 10 days. Cells were fixed and then stained with crystal violet, and photographed under the microscope (Olympus). For EdU (5-ethynyl-2’-deoxyuridine) staining, cells were treated with 50 μM EdU (RiboBio, Guangzhou, China). Cells were fixed, permeabilized, and then incubated with specific antibody against EdU (1:500; Abcam). Cells were then subjected to apollo staining reaction and observed under the microscopy (Olympus) according to previous research [13].

### Wound healing assay

TPC-1 and Cal62 were seeded in 6-well plate two days after transfection and then scratched by pipette tip. Twenty-four hours later, the wound gaps were photographed under microscope (Olympus) according to previous research [15].

### Transwell assay

TPC-1 and Cal62 in serum-free RPMI-1640 medium were added into the upper chamber of Matrigel-coated well (Corning, Tewksbury, MA, USA). RPMI-1640 medium containing 20% fetal bovine serum was added to the lower chamber. Twenty-four hours later, methanol-fixed and then crystal violet-stained cells were photographed under light microscope (Olympus) according to previous research [15].

### Western blot

Protein of tissues and cells were segregated and then transferred onto a nitrocellulose membrane. Membrane was blocked and then incubated with primary antibodies: anti-CEMIP and anti-GAPDH (1:2000), anti-STAT3 and anti-p-STAT3 (1:2500), anti-AKT and anti-p-ANT (1:3000), anti-p65 and anti-p-p65 (1:3500). The membranes were then incubated with secondary antibodies (1:4000) and subjected to chemiluminescence reagent kit (Beyotime Biotechnology, Beijing, China). All the proteins were purchased from Abcam (Cambridge, MA, USA) according to previous research [15].

### Mouse xenograft assay

The study was approved by the Ethics Committee of First Affiliated Hospital of Harbin Medical University (Approval No. IRB-AF/SC-04/01.0) and in accordance with the National Institutes of Health Laboratory Animal Care and Use Guidelines. Total of ten female BALB/c nude mice were divided into two groups: sh-NC (N = 5) and sh-CEMIP#1 (N = 5). TPC-1 cells with stable down-regulation of CEMIP or the negative control (sh-NC) (Genepharma, Shanghai, China) were subcutaneously injected into nude mice. The tumors were measured every week and the volume was calculated five weeks after the injection according to previous research [15].

### Immunohistochemistry

Tumor tissues from mice were fixed with 10% formalin, embedded in paraffin, and then sliced into 4-µm-thick sections. Sections were incubated in 3% H2O2, immersed in Tris-EDTA buffer containing 0.05% Tween 20, and blocked in 0.3% goat serum and 5% dry milk. Specific antibodies against CEMIP, p-AKT, p-STAT3, and pp-p65 (Abcam) were used to incubate with the sections, and the sections were then treated with horseradish peroxidase-conjugated goat anti-rabbit IgG secondary antibody (Abcam). The sections were examined under a light microscope (Olympus, Tokyo, Japan).

## Statistical analysis

All the data were expressed as mean ± SEM and analyzed by student’s *t* test or one-way analysis of variance for the statistical analysis between groups. *p* < 0.05 was considered as statistically significant.

## Results

### CEMIP was elevated in PTC

**Figure 1.**
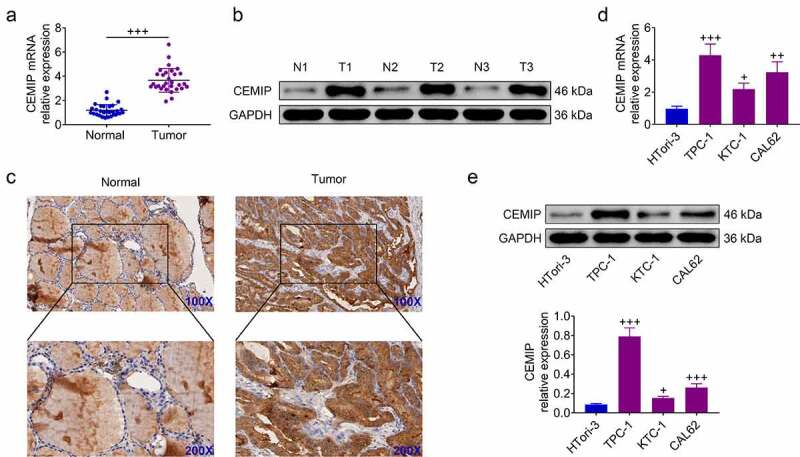
CEMIP was elevated in PTC. (a) CEMIP mRNA was upregulated in PTC tissues. (b) CEMIP protein was upregulated in PTC tissues. (c) Elevated expression of CEMIP in PTC tissues. (d) CEMIP mRNA was up-regulated in PTC cells. (e) CEMIP protein was up-regulated in PTC cells. +, ++, +++ vs. normal tissues or HTori-3, *p* < 0.05, *p* < 0.01, *p* < 0.001.

To investigate the role of CEMIP in PTC, expression level of CEMIP was firstly evaluated. CEMIP was upregulated in the tumor tissues (Figure 1(a,b)). Moreover, immunohistochemistry also confirmed the elevated expression of CEMIP in the tumor tissues (Figure 1(c)). PTC cells (TPC-1, KTC-1, CAL62, K1) showed higher expression of CEMIP than Htori-3 (Figure 1(d,e)).

### CEMIP contributed to PTC cell proliferation

**Figure 2.**
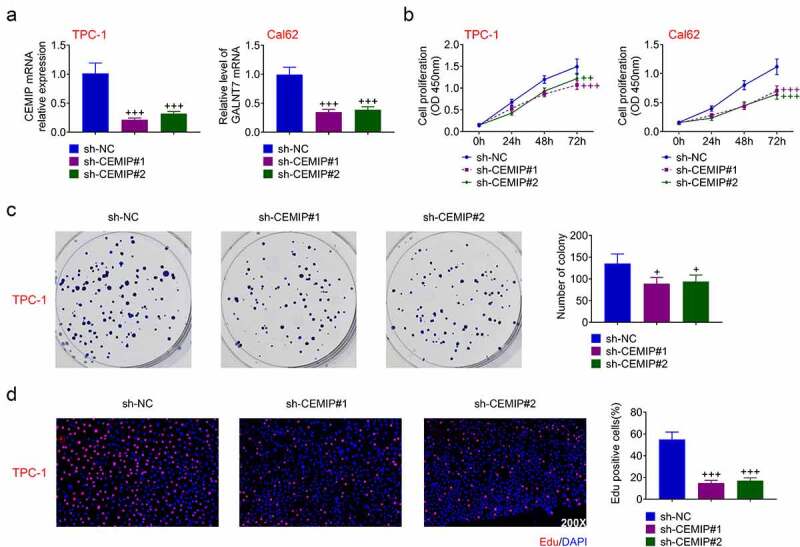
CEMIP contributed to PTC cell proliferation. (a) Silence of CEMIP decreased mRNA expression of CEMIP. (b) Silence of CEMIP reduced cell viability. (c) Silence of CEMIP suppressed cell colony numbers. (d)Silence of CEMIP reduced cell proliferation. +, ++, +++ vs. sh-NC, *p* < 0.05, *p* < 0.01, *p* < 0.001.

Effect of CEMIP on PTC progression was assessed through loss of functional assays. Transfection with sh-CEMIP#1 or #2 decreased mRNA expression of CEMIP (Figure 2(a)) and reduced cell viability of TPC-1 and Cal62 cells (Figure 2(b)). Moreover, proliferation of TPC-1 and Cal62 cells was suppressed by silencing of CEMIP (Figure 2(c)), and knockdown of CEMIP reduced the number of EdU positive cells (Figure 2(d)), demonstrating anti-proliferative effect of CEMIP silencing on PTC.

### CEMIP contributed to PTC cell invasion

**Figure 3.**
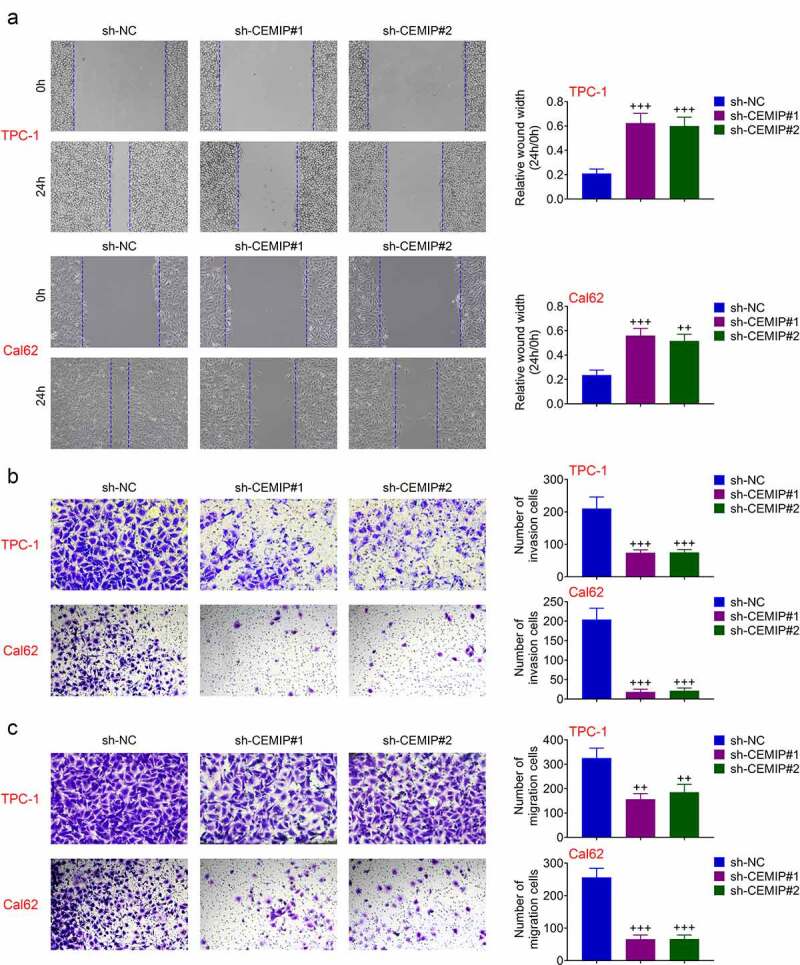
CEMIP contributed to PTC cell invasion. (a) Silence of CEMIP suppressed cell migration. (b) Silence of CEMIP suppressed cell invasion. (c) Silence of CEMIP reduced migration numbers of TPC-1. +++ vs. sh-NC, *p* < 0.001.

Effect of CEMIP on PTC metastasis was also assessed. Migration of TPC-1 and Cal62 cells was repressed by silencing of CEMIP (Figure 3(a)). Transfection with sh-CEMIP retarded cell invasion of TPC-1 and Cal62 cells (Figure 3(b)), and reduced migrated cell numbers (Figure 3(c)), indicating the anti-invasive effect of CEMIP silencing on PTC.

### CEMIP contributed to activation of STAT3/AKT/NF-κB pathway through up-regulation of PDK4

**Figure 4.**
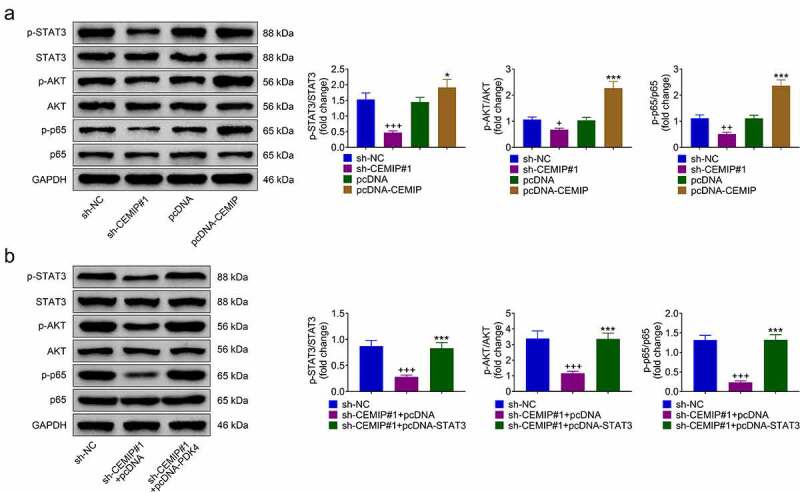
CEMIP contributed to activation of STAT3/AKT/NF-κB pathway through up-regulation of PDK4. (a) Transfection with sh-CEMIP #1 reduced protein expression of p-STAT3, p-AKT and p-p65, and transfection with pcDNA-CEMIP enhanced the protein expression in TPC-1. (b) Over-expression of PDK4 attenuated CEMIP silence-induced decrease of p-STAT3, p-AKT and p-p65. +, ++, +++ vs. sh-NC, *p* < 0.05, *p* < 0.01, *p* < 0.001. *, *** vs. pcDNA or sh-CEMIP #1 + pcDNA, *p* < 0.05, *p* < 0.001.

Underlying mechanism involved in CEMIP-mediated PTC was evaluated. Transfection with sh-CEMIP #1 or pcDNA-CEMIP reduced protein expression of p-STAT3, p-AKT and p-p65 in TPC-1 (Figure 4(a)). However, p-STAT3, p-AKT, and p-p65 were enhanced in TPC-1 transfected with pcDNA-CEMIP (Figure 4(a)). Over-expression of PDK4 attenuated CEMIP silencing-induced decrease in p-STAT3, p-AKT, and p-p65 (Figure 4(b)), revealing that knockdown of CEMIP suppressed PDK4-mediated activation of STAT3/AKT/NF-κB pathway in PTC.

### CEMIP/STAT3/AKT/NF-κB contributed to PTC progression

**Figure 5.**
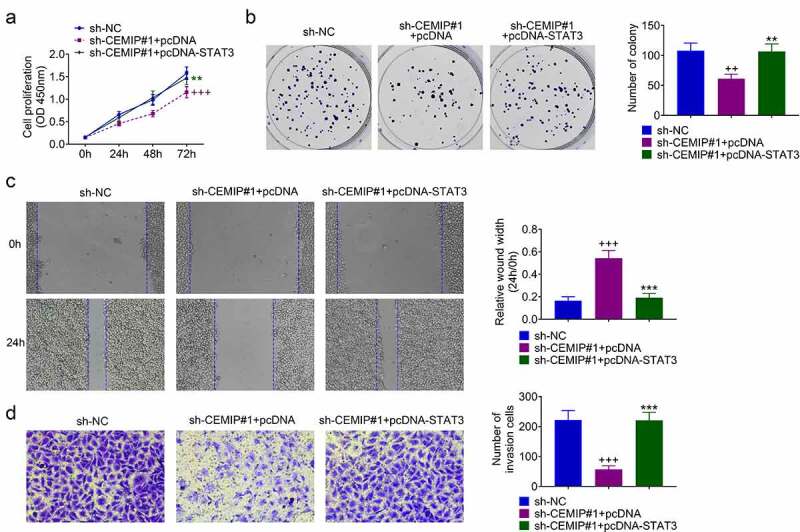
CEMIP contributed to PTC progression through activation of STAT3/AKT/NF-κB pathway. (a) Over-expression of STAT3 attenuated CEMIP silence-induced decrease of cell viability. (b) Over-expression of STAT3 attenuated CEMIP silence-induced decrease of cell proliferation. (c) Over-expression of STAT3 attenuated CEMIP silence-induced decrease of cell migration. (d) Over-expression of STAT3 attenuated CEMIP silence-induced decrease of cell invasion. ++, +++ vs. sh-NC, *p* < 0.01, *p* < 0.001. **, *** vs. pcDNA or sh-CEMIP #1 + pcDNA, *p* < 0.01, *p* < 0.001.

TPC-1 was cotransfected with sh-CEMIP #1 and pcDNA-STAT3 to investigate the role of CEMIP/PDK4/STAT3/AKT/NF-κB axis in PTC progression. sh-CEMIP #1-induced decrease in cell viability (Figure 5(a)) and proliferation (Figure 5(b)) in TPC-1 were restored by STAT3 over-expression. Moreover, STAT3 over-expression attenuated CEMIP silencing-induced suppression of cell migration (Figure 5(c)) and invasion (Figure 5(d)). These results revealed that silencing of CEMIP repressed PTC progression through inactivation of STAT3/AKT/NF-κB pathway.

### CEMIP contributed to tumor growth of PTC

**Figure 6.**
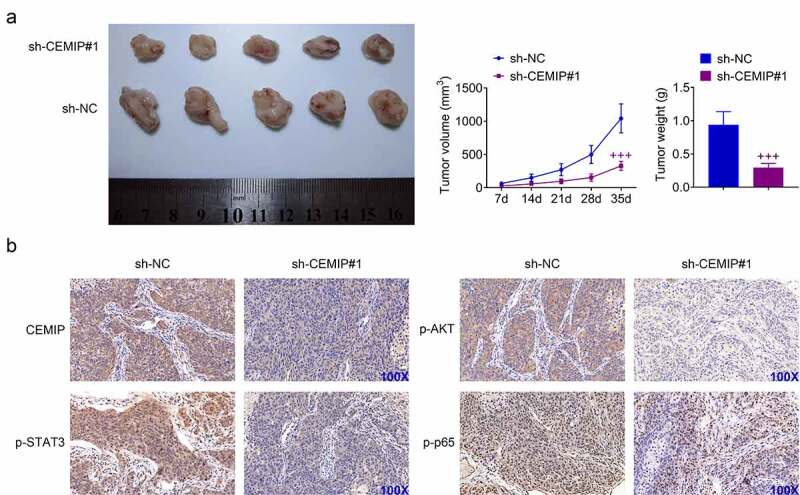
CEMIP contributed to tumor growth of PTC. (a) Intratumoral injection with sh-CEMIP#1 suppressed tumor growth of PTC through decrease of tumor volume and weight. (b) Immunohistochemical analysis showed down-regulation of CEMIP, p-AKT, p-STAT3 and p-p65 in mice injected with sh-CEMIP#1. +++ vs. sh-NC, *p* < 0.001.

Effect of CEMIP on tumor growth of PTC was investigated. TPC-1 with stably silencing of CIMIP were inoculated into nude mice to investigate the role of CEMIP in PTC growth. Mice with intratumoral injection with sh-CEMIP#1 showed smaller tumors than mice injected with sh-NC (Figure 6(a). The tumor volume and weight were reduced with injection of sh-CEMIP#1 (Figure 6(a)). Moreover, immunohistochemical analysis showed down-regulated CEMIP, p-AKT, p-STAT3, and p-p65 in mice injected with sh-CEMIP#1 (Figure 6(b)). These results revealed that silencing of CEMIP suppressed xenograft tumor growth of PTC.

## Discussion

Extracellular hyaluronan modulates cell differentiation, and proliferation, thus contributing to invasion of human cancers [16]. Hyaluronan was considered as a prognostic biomarker of differentiated thyroid carcinoma [16]. Since CEMIP has been found to catalyze the catabolism of hyaluronan, and was identified as therapeutic target of cancers through regulation of tumorigenesis [4], the role of CEMIP in PTC progression was then investigated.

High expression of CEMIP in both carcinoma tissues and cells of papillary thyroid was verified in this study. Diagnostic role of CEMIP has been reported in pancreatic cancer [17]. The correlation between CEMIP expression and the clinical characteristics should be explored to investigate the prognostic and diagnostic roles of CEMIP in PTC.

CEMIP functioned as a metastatic promoter in small cell lung cancer [18]. Knockdown of CEMIP reduced cell viability of PTC, and suppressed the proliferation, migration, and invasion. Epithelial-to-mesenchymal transition has been shown to promote metastasis of PTC [19], and CEMIP promoted the epithelial-to-mesenchymal transition of gastric cancer [20]. Therefore, CEMIP might also promote epithelial-to-mesenchymal transition of PTC.

CEMIP promoted activation of Wnt/β-catenin/Snail [21] and MEK1–ERK1/2 [22] during progression of tumors. STAT3 was activated by CEMIP to facilitate for proliferation and migration of breast cancer [23]. Activation of STAT3 pathway was implicated in progression of thyroid cancer [24]. This study showed that p-STAT3 was reduced by knockdown of CEMIP in PTC, while enhanced by over-expression of CEMIP. STAT3 over-expression attenuated CEMIP interference-induced decrease in cell viability, proliferation, migration, and invasion of PTC cells, suggesting that CEMIP contributed to PTC progression through activation of STAT3 pathway.

CEMIP promoted PDK4 to enhance the metabolic reprogramming and induce prostate cancer cell metastasis [12]. Blockade of PDK4 suppressed activation of STAT3/AKT/NF-κB pathway to inhibit the cancer stem cell characteristics and glycolysis of ovarian cancer [25]. Inhibition of STAT3 eliminated activities of AKT and NF-κB [26], and STAT3/AKT/NF-κB was activated to repress cell apoptosis and promote cell proliferation of prostate cancer [27]. Protein expressions of p-AKT and p-p65 were also reduced by knockdown of CEMIP, and enhanced by over-expression of CEMIP in PTC. CEMIP silencing-induced decrease in p-STAT3, p-AKT, and p-p65 in PTC cells were restored by over-expression of PDK4, indicating that CEMIP promoted PDK4 to activate STAT3/AKT/NF-κB pathway in PTC.

Our study suggested that CEMIP might be a novel oncogenic gene in PTC. Knockdown of CEMIP suppressed PTC cell growth and metastasis through PDK4-mediated inactivation of STAT3/AKT/NF-κB. The meaningful results might provide potential target for PTC.
